# Movement and sociodemographic predictors of zoonotic and human hookworm infection in rural Madagascar

**DOI:** 10.21203/rs.3.rs-11295240/v1

**Published:** 2026-10-09

**Authors:** Georgia Titcomb, Kayla Kauffman, Tyler Barrett, Randall Kramer, Peter Mucha, Michelle Pender, Jean Yves Rabezara, Prisca Rahary, Toky Randriamoria, Voahangy Soarimalala, Charles L. Nunn

**Affiliations:** Colorado State University; Stanford University; Duke University; Duke University; Dartmouth College; Duke University; ĽUniversité Antsiranana; Association Vahatra; Association Vahatra; Association Vahatra; Duke University

**Keywords:** Necator americanus, Ancylostoma ceylanicum, DNA metabarcoding, Human–animal interactions, Land use patterns, GPS tracking, Spatial epidemiology, One Health

## Abstract

Hookworms infect half a billion people worldwide, disproportionately impacting those living in poverty. Previous research has focused on sociodemographic predictors of infection, while less is known about how human land use influences infection. Moreover, recent molecular research has revealed that humans are frequently infected by multiple hookworm species that likely differ in their transmission patterns. In this study, we combined DNA metabarcoding of human and domesticated animal parasites with fine-scale GPS movement data to investigate how hookworm infection relates to people's land use and sociodemographic characteristics in three villages in rural Madagascar. We found high hookworm infection prevalence (52% in women and 69% in men; n = 881). *Necator americanus* predominated (53% prevalence), but the zoonotic hookworm *Ancylostoma ceylanicum* was also common (20% prevalence). Domesticated animals hosted several parasite species typically absent in humans. However, the zoonotic hookworm *A. ceylanicum* was highly prevalent in dogs (67–88% across villages). Consistent with previous findings, men had double the odds of *Necator americanus* infection than women, and prevalence was higher in those with lower material wealth (64% prevalence in the bottom quartile versus 47% prevalence in the top quartile). Zoonotic hookworm infections were more common in men than women (OR = 1.54), those with less formal education, and those reporting close contact with dogs (OR = 1.61). Regarding land use by people, we found that larger home ranges and greater use of vegetated areas outside villages was linked with increased *N. americanus*, but not *A. ceylanicum*, infection risk. Network analyses revealed that infected individuals were more strongly connected to each other than to uninfected individuals on networks of shared vegetated patches outside of the village. However, in one village with low levels of vegetation cover, we found significant spatial associations between people’s dwellings and human hookworm infections, illustrating the importance of ecological context for hookworm transmission. These findings underscore the importance of integrating fine-scale land use patterns with sociodemographic risk factors to better understand and mitigate human and zoonotic hookworm transmission.

## Author Summary

Hookworms are some of the most common intestinal parasites worldwide and have substantial health impacts on communities facing poverty. Past studies have shown how socioeconomic factors are associated with infection, but we have little understanding of how these characteristics relate to people’s daily movements and interactions with the landscape in ways that influence transmission. Recent research has also suggested that zoonotic hookworms may be more common than previously thought, as traditional screening methods cannot differentiate among hookworm species that may have different transmission routes. In this study, we addressed this knowledge gap by combining parasite DNA sequencing with GPS tracking of fine-scale human movements to examine hookworm infection patterns in rural Madagascar, where hookworms are highly prevalent and understudied.

We found that people were infected by two hookworm species: the human parasite *Necator americanus* and the zoonotic parasite *Ancylostoma ceylanicum*, which was also highly prevalent in dogs. Men, individuals with less wealth, and those who used vegetated areas outside the villages were more likely to be infected. Our findings reveal parasite communities in humans and their domesticated animals and demonstrate that both sociodemographic variables and movements across the landscape are associated with infection, providing spatial insight for prevention strategies.

## Introduction

Approximately 500 million people worldwide are infected with hookworms (1). Hookworm infections account for more than 4 million disability-adjusted life years and up to $138.9 billion in economic costs, placing hookworm-associated diseases on par with several higher-profile infections, such as dengue and tuberculosis (2). Moreover, hookworm infections disproportionately affect individuals in poverty (3, 4) and may contribute to cognitive impairments (5) that perpetuate ‘poverty traps’ (3). While individual infections can be treated with anthelmintics, deworming interventions are often unsuccessful because people are quickly re-infected by larvae that persist in the environment (4, 6), highlighting the public health importance of reducing hookworm exposure to control infections. While several sociodemographic factors have been connected to variation in hookworm prevalence, little is known about the underlying behaviors that ultimately determine exposure risk. Disentangling the connections between demographic factors and exposure risks is important for devising interventions that reduce exposure efficiently in high-risk groups.

Two major hookworm species infect humans: *Necator americanus* and *Ancylostoma duodenale*. Humans can also be infected by zoonotic species that circulate in domesticated animals, namely dogs (e.g., *Ancylostoma ceylanicum a*nd *Ancylostoma caninum*). New molecular tools have revealed that *A. ceylanicum* is an emerging parasite in humans, and that humans can act as definitive hosts (7). Across hookworm species, transmission occurs when parasitic larval stages penetrate the skin or are ingested and enter the circulatory system, eventually reaching the intestines where they reproduce sexually and expel eggs with host feces (8). Human infections typically last multiple years unless treated with anti-parasitic drugs (albendazole and mebendazole) (1). Parasite eggs expelled into warm, wet soils develop into larvae that remain infectious in the environment for multiple weeks under ideal conditions but are susceptible to sunlight and desiccation (9).

While clinical symptoms and worm life cycles are similar for human and zoonotic hookworm infections, differences in transmission may lead to different sociodemographic patterns of infection. Prior research has documented numerous sociodemographic predictors of human hookworm infection, including gender (10), age (10, 11) (12), and education (10). However, it is unclear whether these variations are driven by differences in immunity (13) or differences in exposure (11) (14). Despite strong and consistent sociodemographic associations with hookworm infection, there is little fine-scale empirical data linking sociodemographic characteristics to human movement patterns and risk of hookworm exposure. At least one study linked occupational risks (using human feces as fertilizer) to hookworm infection in Vietnam (15), and another found slight spatial patterns in hookworm prevalence in children in Cote d’Ivoire (16). However, the spatial scale (1km) and georeferencing only to school location constrained the predictive power of this analysis.

Pinpointing transmission of hookworms is exceptionally challenging. Historically, most human infections were considered to involve *Necator americanus* and *Ancylostoma duodenum*, with zoonotic infections being relatively rare (7). This understanding has changed with the advent of molecular techniques that can differentiate hookworm species in stool, revealing that zoonotic hookworm infections are common (e.g., (17, 18)). These findings complicate understanding of sociodemographic risk factors because exposure risks may differ substantially across zoonotic and non-zoonotic parasites given differences in transmission and exposure patterns. For human-hookworm infections, transmission dynamics are thought to be driven by open defecation and lack of shoes. However, in the case of *A. ceylanicum*, zoonotic transmission patterns may fundamentally differ because areas where dogs defecate are likely to differ from areas where humans defecate. These differences have public health importance, as they determine whether interventions should focus on human or animal movement and defecation patterns.

Hookworm infections are a significant health problem in Madagascar, where high prevalence (up to 45%) has been reported (19). Prior work in rural Malagasy villages has suggested that infections are more common in males (20), those with less education (20, 21), older individuals (21), and in people living further from a major road (21), but little is known about how hookworm infections relate to exposure risk across space. While there are ongoing mass anti-parasite drug administration programs in Madagascar, these efforts often target schoolchildren and will likely have limited impact on transmission among adults and ongoing exposure. Thus, limited sanitation and parasite control among adults make population well suited for asking investigating how sociodemographic factors and shared land use shape transmission of hookworms.

In this study, we screened for multiple worm infections in humans and their domesticated animals in three rural villages in Madagascar using DNA metabarcoding. We then considered how sociodemographic characteristics influence exposure to human and zoonotic hookworm, including predictors of co-infection, hypothesizing that men would have higher infection prevalence than women, with this pattern partially explained by men spending more time in vegetated areas outside the village where they have greater exposure to infectious larvae due to open defecation and improved parasite survival. We expected a similar pattern for individuals with less formal education and lower socioeconomic status. For zoonotic hookworms, we hypothesized that participants with greater contact with dogs would have greater risk of infection, while other sociodemographic predictors would be less informative because exposure to dog feces is more evenly distributed across the environment.

We also investigated how human hookworm infection varies with fine-scale human movement data and networks of shared land use in different habitats. We hypothesized that demographic differences in human hookworm infections would be echoed in movement data, and that infected individuals would be more likely to be connected to other infected individuals on networks representing shared land use. Through this research, we aim to expand understanding of risk factors for hookworm infection and provide new insights into disease control.

## Methods

### Ethics statement

The Duke University Institutional Review Board (Duke IRB Number 2019 − 0560) and the Madagascar Ministry of Public Health’s Ethics Committee for Biomedical Research (Permit Number 114 MSANP/SG/AGMED/CERBM) approved the study protocols. Participants provided oral consent for surveys and written consent for GPS trackers and fecal sampling. Participants consenting to the GPS aspect of the study were provided with information about the technology and data use (22).

## Study design and setting

Research was conducted from 2019–2022 in the SAVA region of Northeast Madagascar in three different communities surrounding Marojejy National Park. Hereafter, these communities are referred to as village A, village M, and village S. In each village, participants were enrolled in the study over three seasons (dry, wet, and transitional) for 3–6 weeks following methods described in (22–24). Village M was not surveyed during the transitional season due to the COVID-19 pandemic. Study participants were recruited using snowball sampling (25). This sampling approach was initiated with a set of individuals who owned agricultural land in each of the three study villages. We then enrolled people they named as individuals with whom they spend their free time, exchange farmwork, or exchange food. The estimated population in each village was 1900, 2700, and 900 for villages A, M, and S, respectively.

## Surveys

All participants (n = 1278) completed a survey administered in the local Malagasy dialect by a trained research team. Responses were recorded on a Samsung tablet using Qualtrics software (Qualtrics, Provo, Utah). During the survey, participants could elect to provide fecal samples for parasite screening (n = 885) and to wear a GPS tracker that recorded their location every three minutes for one to five weeks (n = 678 participants with fecal samples + GPS). All participants agreed to participate in a health, demographic, and animal contact survey. Participants were compensated with mobile phone credits. Participants also consented to fecal sampling of their domesticated animals (n = 207). When individuals owned more than one animal, the participant selected one animal for sampling and answered questions about that animal’s health. Comparison with survey data showed that sampled animals were broadly representative of the species present in the villages, except for cats, which were the fourth-most common animal, but which were not included in this study due to logistical challenges in locating and sampling feces.

Each participant reported their age, gender, and highest level of education, and close contact with different domesticated animal species in the prior year, defined as having the animal as a pet within the home, handling, rearing, sharing water, or eating the animal, as well as observing the animal’s feces near food, or being scratched or bitten by the animal. We also used principal component analysis to investigate durable goods and household material wealth, as described in detail in (22). First, we asked participants about the material they used to construct their house, including floors (dirt, locally acquired plants [bamboo, ravinala], or cement), walls (locally acquired plants, mud, compacted earth, wood planks, bricks, metal sheets, or cement), and roofs (locally acquired plants, metal sheets, or cement). We also asked participants about the type of latrine they owned (none, pit latrine, or improved pit latrine). Second, we asked participants to report whether their household owned any of nine durable goods, including cars, motorcycles, bicycles, refrigerators, computers, generators, televisions, radios, and cell phones.

## Fecal Sampling

Participants were provided with fecal sampling kits, from which approximately 1g of stool was preserved in either Nucleic Acid Preservation buffer (NAP, (26)) or DNA Shield (Zymo). Different buffers were used due to supply restrictions as a complication of the SARS-CoV-2 pandemic. Buffer type had no significant effect on read abundance from DNA sequencing or prevalence of any parasite (n = 881, *T*_*reads*_ = −0.78, *p* = 0.44; $\chi_{\text{N. americanus}}^{2}=0.031$, *p* = 0.86; $\chi_{\text{A. ceylanicum}}^{2}=2.56$, *p* = 0.11). We extracted DNA from stool using Zymo Quick-DNA Fecal/Soil Microbe Miniprep kit (cat: D6010) following manufacturer protocols.

## Metabarcoding

We amplified multiple species of nematode DNA in human and animal samples using 8-nt dual-indexed barcoded ITS-2 primers for strongylid nematodes (27). Barcodes were designed to have a Hamming distance of at least 4 and were checked for self-dimerization and hairpin issues.

DNA extracts from 885 human fecal samples and 209 cows, pigs, and dogs were amplified (minimum DNA concentration = 1ng/uL). Separate libraries were prepared for each of the three different villages. PCR mixtures included 2.5mM MgCl2 and 1X Amplitaq Gold II Buffer (Applied Biosystems, Foster City, California, USA), 200uM each dNTP, 0.17 mg/mL BSA (New England Biolabs), 0.4% DMSO (Sigma Aldrich), 0.2uM each primer, 0.38U Amplitaq Gold polymerase (Applied Biosystems), and 2uL DNA extract in 15uL reactions. Thermocycling conditions included initial denaturing at 95°C for 10min, followed by 35 cycles of 95°C for 15s, 55°C for 30s, and 72°C for 40s, with a final 5min extension at 72°C. DNA concentration in amplified products was measured and normalized to a standard concentration (7ng/uL) before pooling and purification using MinElute columns (Qiagen, Hilden, Germany). The pooled library was multiplexed with additional samples and prepared for sequencing using a PCR-free approach at the UC Davis Genomics Core. Sequencing was performed at UC Davis using Illumina MiSeq (24M reads, v3 chemistry 300×2). We included a PhiX spike-in of 15% per run and re-sequenced the library three times to increase read depth. For any sample with fewer than 500 reads for this locus and a concurrently-analyzed eukaryome primer set (28), we repeated amplification and sequencing (n = 35).

We then demultiplexed individual samples and combined reads from separate runs using cutadapt (v 3.4), setting the maximum error rate to zero. We performed subsequent filtering steps using the *dada2* workflow in R (29). Briefly, this involved filtering and trimming amplicons (minLen = 100, removing PhiX), learning errors, dereplication, inferring ASVs using the pseudo-pooling method, merging pairs, removing chimeras, merging sequence tables for separate libraries, and performing taxonomic assignment using the assignTaxonomy function (min boot = 50) with the nemabiome reference database (v1.6.0) (30). To control for potential sequencing errors, we removed all sequences that accounted for less than 1% of a sample’s total read count. Samples with at least 500 reads were considered positive for strongylid nematode DNA, while samples with fewer than 500 reads were considered negative (Appendix S1, Figure S1).

We excluded four samples from the original 885 human samples: one was identified as cow feces based on concurrent 16S microbiome analyses, one failed to amplify across multiple primer sets, and two were repeat samples of the same two individuals, resulting in a final dataset with 881 human samples and 209 domesticated animal samples.

### GPS analysis

GPS devices (iGot-U 120; Mobile Action, New Taipei City, Taiwan) were provided to participants for a one-week period, after which participants could decide to continue wearing the device for up to 6 additional weeks. GPS data were logged every three minutes, with cleaned data used to estimate participants’ utilization distributions, as described in Kauffman et al. (23). Briefly, we excluded data from the day of deployment to avoid measuring movements influenced by study enrollment. Erroneous points were detected and removed based on speed and heading. We excluded days when a participant appeared to not wear the device using a daily minimum convex polygon size threshold of < 1 ha. To confirm lack of movements in these cases, we visualized the days’ GPS trajectory to confirm the device was stationary. Because a large proportion of utilization distributions were comprised of time spent sleeping at home when hookworm transmission is likely to be negligible, we filtered data to typical active hours (6am-8pm) only. We estimated utilization distributions from cleaned GPS data at a 10m^2^ resolution using a dynamic Brownian bridge movement model (31). We used the 95% utilization distribution (i.e., the range where each individual spends 95% of their time) to describe relative time spent in different land cover classes and spatial overlap between participants.

## Land cover classification

We used ArcGIS Pro to classify the landscape surrounding each of the three villages by creating a composite image from twelve Sentinel-2 images that represented the three different seasons corresponding to GPS movements over the 3-year study period. We then created training samples by classifying land into 7 types: semi-intact forest, secondary forest fragments, brushy/open habitat, bare ground, rice field, water, and village. We ran a support vector machine model to classify the rest of the landscape and used the majority filter tool to clean up edges. Finally, we manually inspected and corrected any incorrect grid cells that intersected with areas of high use based on overlapping utilization distributions. The time participants spent in each landcover type was determined using the methods described in (23). In brief, we first cleaned GPS data to remove erroneous points and days that participants did not wear the device, and to exclude excursions that do not represent typical movements. We then calculated individuals’ utilization distributions (UD) during typical active hours (06:00 to 19:59 GMT + 3) using a dynamic Brownian bridge movement model (31) and combined this UD dataset with land classifications at the 30×30m pixel level (Fig. 1).

## Analysis

We used a combination of generalized linear modeling and network tests to explore how sociodemographic and movement predictors related to both human and zoonotic hookworm infections (Fig. 2).

### Socio-demographic analyses

Our first objective was to understand how sociodemographic characteristics corresponded to infection patterns. We used generalized linear models with a binomial error structure to explore correlates of hookworm infection for *N. americanus, A. ceylanicum*, and co-infections. We built models using several hypothesized covariates of infection: gender [male or female], age, school level [none, primary, secondary, or higher] translated to four integer levels, wealth defined using the first principal component of material goods possession and the first principal component of house materials as described in (22), and dog contact. We also checked for non-independence among households by assigning a household ID to participants who lived within 5m of each other and running a generalized linear mixed model with household ID as a random effect. Given that AICc values were lower for GLMs than GLMMs for both single worm models, and that residuals in the coinfection GLMM violated assumptions tested using the *DHARMa* package (32), we report all results from GLMs only.

We fit full models including all hypothesized covariates and used the *dredge* function in the *MuMIn* package (33) to compare all model subsets, ranking them based on AICc values. We performed model averaging on the top set of models (ΔAIC ≤ 2) to identify top covariates for each parasite model.

### Movement analyses

Our second objective was to understand if land use patterns corresponded to infection status. To identify specific drivers of variation in movement by infection, we performed principal component analysis on the multivariate dataset of participants’ utilization distributions across the seven different land classifications. We then used a binomial generalized linear model to investigate whether the first two principal components explained variation in infection across individuals for both parasite species. We also performed a complementary permutational MANOVA to test whether utilization distributions across land classes varied by parasite infection (Appendix S6).

To investigate how infection covaried with shared use of different habitats, we built a suite of networks based on people’s utilization distributions. First, we built a bipartite network for each of the dominant habitat classifications in each village (secondary forest, brushy regrowth, rice, any area outside the village, and village), in which individuals were connected to grid cells, with edges weighted by their utilization distribution in each grid cell as described in (23). We then projected each network to a unipartite network that connected participants to each other via edges weighted by the number of 30m^2^ gridcells present in the 95% utilization distribution of both individuals. To incorporate time spent in gridcells, we built a second set of networks in which edges were weighted by the sum-product of the utilization distributions (i.e., the integral of the joint probability distributions) (34) for each dyad. We also created a unipartite house distance network based on the distance between participants’ houses in each village, and we included the friendship network to explore whether long-term social relationships also corresponded to infection patterns. Spatial networks were only weakly correlated with the friendship network that informed participants’ enrollment via snowball sampling, while land use networks tended to be more strongly correlated to each other (Appendix S2, Figure S2).

For each network, we labeled nodes as either infected, uninfected, or untested (and thus unknown). For both hookworm species, we used the network k-test of independence (35) to investigate whether the network predicted patterns of infection. The standard implementation of the k-test is based on network degree centrality and requires binary (present-absent) edges without weights indicating the amount of interaction between pairs of individuals. Because many networks were dense (i.e., nearly all individuals on the village network were connected to each other because most people spent time in the same area at least once), we created three sub-networks using different thresholds to define edges, with edges representing the top 10, 20, and 30th percentile of edge weights for each network, and distance thresholds of 25m, 50m, and 75m for the house networks. We also used a variant on this method that used weighted edges (strength) to investigate the hypothesis that infected individuals were more strongly connected to other infected individuals on networks representing shared use of habitats. Since k-test results were similar using both approaches, we present k-test results on network strength here and provide the standard k-test results in the supplement.

## Results

The number of participants and associated sociodemographic and land use characteristics used for analyses are provided in Table 1.

### Prevalence and distribution

*Necator americanus* prevalence in our sample of people was 53% across all individuals (n = 881) but varied across the three villages: (village A: 61.4%, village M: 46.7%, village S: 46.1%, *χ*^2^ = 20.12, p < 0.001). The zoonotic hookworm *A. ceylanicum* was found in 20.4% of individuals and prevalence varied among villages (A: 12.9%, M: 23.4%, S: 30.0%, *χ*^2^ = 30.54, p < 0.001; Fig. 3). Across all villages, 12.9% of people were coinfected by *N. americanus* and *A. ceylanicum*. Infection prevalence did not vary significantly by recruitment chain in any village (Appendix S5), indicating that recruitment structure likely had limited influence on village-level prevalence estimates. Infections were non-independent in village M, where individuals infected with one parasite had higher odds of infection with the other parasite (OR: 6.11, p < 0.001). However, in village S, the odds ratio was 1.67 and only marginally significant (p = 0.056), and in village A, the odds ratio was 1.08 and not significantly different from 1 (p = 0.70), suggesting that infection with zoonotic hookworm was not associated with human hookworm infection. We detected only two incidental infections in people with other strongylid worm species, *Trichostrongylus colubriformis* and *Trichostrongylus axei*.

Domesticated animal infections are reported in Fig. 4. Cows were infected by the common livestock worms *Trichostrongylus axei* (15–38% prevalence across villages), *Cooperia spatulata* (15–26%), and *Haemonchus placei* and *H. similis* (5–22%). There were incidental detections of *A. ceylanicum, Oesophagostomum radiatum*, and *Nippostrongylus brasiliensis*, which are species that do not commonly infect cows, but which are prevalent in other species in the system. Dogs showed a high prevalence of *Ancylostoma ceylanicum* (67–88% across villages) as well as the related dog-specific hookworm, *Ancylostoma caninum* (22–50%). For pigs, two nodular worms in the genus *Oesophagostomum* (*O. quadripinulatum* and *O. dentatum*) were common (13–43% prevalence), as well as a pathogenic stomach worm, *Hyostrongylus rubidus*, which was found in high prevalence in villages M and S but was absent from village A. We also detected the presence of worms in the *Ancylostomatidae* family, which has not been extensively documented to have high prevalence in pigs.

### Demographic predictors

Gender and village were the only two predictors with significant model-averaged coefficients across all three models of infection (*N. americanus*, *A. ceylanicum*, and coinfection; Fig. 5). Residents of village A had higher odds of infection with *N. americanus* and lower odds of *A. ceylanicum infection* and coinfection than residents of villages M and S. Infection also varied by season, but this was inconsistent across the two parasite species, as the odds of *N. americanus* infection were highest during the dry season, while odds of *A. ceylanicum* infection were elevated during the transitional season. Additionally, men had higher odds of infection than women in all three models (OR = 1.99, 1.50, and 1.63 for *N. americanus*, *A. ceylanicum*, and the coinfection models, respectively; Fig. 5). Counter to our predictions, age, school level, and wealth were not significant predictors of infection with *N. americanus* or coinfections; however, less schooling was indicative of infection with *A. ceylanicum*. Lower material wealth, as represented by smaller PC1 values, was indicative of infection with *N. americanus* and coinfections; however, house-based wealth was not. While dog infection data was limited and precluded our ability to test for matched infections in individual dogs and people with whom they shared space, any close contact with dogs was associated with increased infection risk with *A. ceylanicum* (OR = 1.61, p = 0.009), but not *N. americanus* nor coinfections. Household dog ownership alone was marginally associated with increased *A. ceylanicum* infection (OR = 1.37, p = 0.09). Component model details and conditional coefficients for averaged models are reported in Appendix S3 and S4.

### Movement and contact predictors

The PERMANOVA of land use revealed that sociodemographic characteristics and infection status explained variation in land use for *N. americanus* but not *A. ceylanicum* (Appendix S6).

The principal component analysis condensed land use to two axes (PC1 and PC2) that explained 22.1% and 15.6% of the variation respectively (Fig. 6A and B). PC1 partitioned village areas from the rest of the landscape, with low values associated with use of the village and high values indicating greater use of secondary fragments and brushy regrowth. PC2 differentiated between bare ground and areas of rice and water. The GLMs of infection status by land use principal components showed a significant positive association between PC1 and *Necator americanus* infection in villages A and M (p = 0.008 and p = 0.046 respectively), but not for village S (p = 0.525; Fig. 6C). PC2 was not associated with *N. americanus* infections. For *A. ceylanicum*, PC1 was positively associated with infections in village S only (p = 0.035), and PC2 was not significantly associated with *Ancylostoma ceylanicum* infections (Fig. 6D).

### Network Results

Network k-tests revealed that hookworm infection was clustered within social networks representing long-term patterns of associations across villages for both parasite species. Specifically, infected individuals were connected to 2–3 times as many infected friends as would be expected by chance (Figure S4). Additional k-tests on networks of landscape use, which were used to identify likely locations of transmission, supported our hypothesis that networks built from movement data can be used to identify transmission pathways in different contexts. In general, results from networks based on overlapping area (sum of total gridcells used for any period of time) and overlapping time (sum product of time spent in the same areas) were correlated in terms of effect size and direction (Spearman’s *ρ*_effect_ = 0.69, *p*_effect_ < 0.001) but not p-value ($\rho_{p_{\text{value}}}=0.14,p_{p_{\text{value}}}=0.51$), indicating consistent signal in landscape-infection interactions, but variation in power and noise. In the binary k-test analysis, networks based on overlapping area were less sensitive to arbitrary thresholds than networks based on overlapping time. Therefore, we discuss results for overlapping area, while reporting all results in Appendix S7.

Networks of land use outside the village were linked to *N. americanus* infection in the two villages (A and M) that had significant tree and scrub cover (Fig. 7A). For these networks – which included individuals with infected, uninfected, and unknown status – the mean strength centrality of infected alters (i.e., infected individuals within one step of an infected focal individual) was 55% higher than would be expected by chance. In village A, this finding was particularly strong for secondary forest fragments and brushy regrowth networks: infected individuals connected to another infected individual had substantially higher strength centrality than expected by chance (secondary forest = 80% increase, brushy regrowth = 70% increase). Strikingly, in both villages, house distance had no relationship to *N. americanus* infection patterns, which is consistent with transmission outside of home areas. In contrast, in village S, where people spend notably more time in land cover dominated by rice fields and village areas (Fig. 1), land area networks outside the village had no relationship to *N. americanus* infection and instead the house distance network predicted infection (infected individuals had 20% more infected individuals within 50m of their homes than would be expected by chance, p = 0.02).

For *A. ceylanicum*, the zoonotic hookworm, we found little relationship between land use networks and infections in villages M and S. However, in village A, where dog populations are smaller, areas outside the village, including the secondary fragment, brushy regrowth, and rice field networks, as well as the village network significantly correlated with *A. ceylanicum* infections but the household network did not (Fig. 7B).

## Discussion

We demonstrated a novel approach for understanding how infection dynamics vary spatially, providing a more mechanistic understanding of transmission risk than available through demographic predictors or environmental sampling alone. Thus, our results support the well-established finding that human hookworm infection risk is heightened in men compared to women, while providing new insights into the mechanisms underlying gender disparities in infection by linking demographic characteristics to transmission-related movements across the landscape. We also highlight the importance and high prevalence of a zoonotic hookworm, *Ancylostoma ceylanicum*, a parasite that has been historically underreported. We connect its transmission to risk factors related to gender, schooling, and dog density, which may inform interventions to mitigate exposure risk.

Results from the sociodemographic analysis revealed higher infection prevalence of both human and zoonotic hookworm in men. This finding is consistent with a systematic meta-analysis reporting that males have 1.43 times the odds of hookworm infection (namely *N. americanus*) compared to females (36). However, many studies rely on medical data that often lack concurrent details on exposure, making it challenging to disentangle whether these gender-based differences are driven by disparities in exposure or immune susceptibility (11). In our study, we observed that men tended to venture farther than women from village centers and the latrines that are available there. This behavioral tendency may increase the likelihood of practicing open defecation, which is a significant risk factor for disease transmission (37). Away from the village, adults are more likely to defecate in locations that offer privacy, such as wooded habitats surrounding rice fields, where people are less likely to wear shoes, and where shading may enhance survival of hookworm larvae (38). In turn, shaded environments may further increase exposure risk by offering refuge from hot conditions.

While comprehensive studies of *A. ceylanicum* infection are limited, prior work has not consistently reported higher infection risk in men than women (17). Instead, behavioral factors such as walking barefoot, poor hand hygiene, frequent contact with dogs and cats, and limited access to latrines are hypothesized predictors of infection (17, 18). This variation in risk factors across studies likely reflects cultural and geographic differences in exposure, such as differences in sanitation infrastructure, occupational activities, and patterns of human-animal interactions.

We found considerable variation in infection across villages, even when those villages were located relatively close to one another (within 20 km). Notably, people living in the rice-dominant village with little vegetation (village S, Fig. 1) experienced different predictors of infection compared to people in the other two villages (A and M; Figs. 6 and 7). Residents of rice-dominant village S spent only about half as much time in ‘high risk’ environments such as secondary fragments and brushy regrowth, compared to those in villages A and M (9–11% vs. 18–27%; Fig. 1). Instead, infection dynamics in village S more closely resembled those of a household network, suggesting that shared latrines within the village represent a more likely source of hookworm transmission than open areas surrounding rice fields. Moreover, villages with greater access to tree and shrub cover (villages A and M) had stronger associations between *N. americanus* infection and human movements in vegetated areas outside the village, potentially due to increased use of these areas for defecation in privacy combined with the favorable conditions these environments provide for hookworm survival, such as increased shade and soil moisture (38).

Variation in and correlations among sociodemographic parameters at the village scale may have occluded some significant associations between infection and other hypothesized factors. For example, household dog ownership was 40% and 66% more common among participants in villages M and S, respectively, than among participants in village A (Table 1), which likely contributed to higher infection levels of *A. ceylanicum* in these locations at the village scale, as compared to an individual scale. Additionally, we found that greater material wealth was correlated with more schooling, potentially explaining why both variables were included in averaged models for *N. americanus* and *A. ceylanicum*, but only one model-averaged coefficient was significant at α < 0.05. Prior studies have demonstrated increased odds of soil-transmitted helminth infections among individuals with lower socioeconomic status (39, 40), including *N. americanus* (3). Educational attainment has been hypothesized to suffer as a consequence of *N. americanus* infection due to its adverse effects on cognitive development and learning in children, perpetuating a ‘poverty trap’ (3, 41, 42). However, considerably less research has examined these relationships for *A. ceylanicum*. The few studies that have examined sociodemographic associations with this zoonotic parasite have found stronger evidence for the effects of exposure – such as contact with animals and lack of footwear – than for sociodemographic predictors (43, 44).

While we observed broad similarities in relationships between sociodemographic variables and infection patterns for the two hookworm species, we found notable differences in patterns of infection and movement. Specifically, infections with the human hookworm *N. americanus* were more strongly associated with networks of shared habitats, whereas infections with the zoonotic hookworm *Ancylostoma ceylanicum* showed little relationship to human land use and movement, except in village A where dog contacts were lower and prevalence in humans was also lower than in the other villages.

We hypothesize that the high prevalence of *A. ceylanicum* in dogs (67–88%) suggests that contact with soil contaminated by dog feces is the primary transmission route in this system, a finding that is supported by documented patterns of increased infection risk among individuals with higher contact rates with dogs (44, 45). Since dog defecation patterns, as opposed to human defecation patterns, are more likely to be randomly distributed across areas where people move, we hypothesize that exposures are more evenly distributed across individuals in a village such that human movement patterns are not a powerful predictor of infection with zoonotic hookworms.

We propose that our finding of multiple significant relationships between human movements and zoonotic hookworm infections in village A may have been driven by a greater degree of shared time and space between humans and dogs in brushy and covered habitats outside the village. These results may also be explained by the lower levels of dog contact in this village: rather than dog-driven transmission, humans may account for a greater share of transmissions such that dynamics resemble those of *N. americanus*. Firmer conclusions on these patterns would require additional data, such as spatial mapping of human and dog defecation sites. However, these results suggest that awareness and control of dog feces in and around village areas will likely reduce village-level prevalence of zoonotic hookworm. These findings highlight the importance of tailoring public health interventions to local contexts, as well as the importance of identifying the species composition of hookworm infections to design effective control strategies.

Our findings connect patterns of human movement and exposure risk with hookworm infection across sociodemographic variables. While our results align with broad sociodemographic patterns reported in the literature, we advance this understanding by explicitly linking infection risk to variation in exposure driven by movements across the landscape. This may be especially advantageous given that environmental sampling for hookworm larvae is expensive and impractical at large scales. While some prior work has accounted for fine-scale spatial differences in explaining infection patterns (46), this is largely based on GPS coordinates of human residences, and not of movements that capture potential exposure locations. Our results show that house coordinates may have limited utility in improving our understanding of exposure risks, depending on the geographic context and disease.

Infections were also associated with the friendship network for both hookworm species. While the friendship network does not provide insight into where exposures occur, it likely captures latent long-term relationships relevant to transmission risk, including potential similarities in behavior that increase or decrease risk. This is supported by the weak but significant correlation (g_cor_ = 0.01–0.06; all QAP p < 0.01; Appendix S2) between the friendship network and land use networks. From an intervention standpoint, the friendship network may guide efficient treatment strategies, while the spatial associations may guide efficient prevention options by identifying high-risk areas where anti-hookworm precautions should be taken.

Our approach allowed us to identify potential locations and implied activities associated with elevated infection risk. In Madagascar, where rice farming and other agricultural activities outside of village areas are integral to daily life and effective preventative interventions, such as shoe-wearing (47), are often impractical due to financial (48), cultural (49), and occupational (50) constraints. Collaborative shoe design that incorporates people’s occupational needs provides a promising solution to this public health issue.

There are several limitations of the study. First, all study participants were adults. Although prior studies have shown increasing hookworm infection risk with increasing age (up to approximately 15 years, (11)), children likely play a substantial role in hookworm infection dynamics. Consistent differences in childhood land use and infection risk compared to the patterns we observed in adults would complicate our conclusions. Additional data needed to separate adult and childhood risk factors include childhood sociodemographic details and locations where children spend significant time and defecate outside the village. Additionally, our results depend on accurate survey responses and assume that samples reflect the individuals surveyed. However, we note that we found only one case of a non-human sample being submitted for testing (out of the > 800 samples tested).

Second, while metabarcoding is increasingly used to characterize the ‘nemabiome’ of a wide array of hosts, it is not often applied to human infections. We used this approach because we were able to simultaneously screen for a wide range of parasites in humans and their domesticated animals, including zoonotic infections. However, *N. americanus* infections are typically diagnosed using fecal egg counts or qPCR, which may have higher sensitivity and specificity than metabarcoding alone. While qPCR on a single sample has been shown to have similar sensitivity as a three-day stool examination using microscopy, repeated qPCRs further improve detection (51), Thus, it is likely that multi-day sampling would increase prevalence estimates reported here. However, we note that hookworm prevalence from our study is substantially higher than that reported from microscopic examination of stool across several regions in Madagascar (52).

Third, our analyses of land use assume that within a season, individuals tend to repeat their weekly movements across the landscape such that these movements represent longer-term land use patterns that are associated with infection dynamics of both species of hookworm. While movement consistency varies across individuals, we found that the volume intersection of week-to-week movement patterns within a subset of individuals was relatively high at 67% (23), indicating that people in the study demonstrate consistent movements, and that their utilization distribution is an accurate representation of their movements over time frames relevant to hookworm infection. An important consideration in our study is that we were unable to analyze individuals’ movement dynamics across seasons. This may be particularly relevant for individuals who use different parts of the landscape across different growing seasons, as they may have been infected during a season prior to fecal sampling, and this may explain the relatively small proportion of variation in land use explained by infection status. However, our analysis illustrates a broad pattern: individuals that used more heavily vegetated areas outside of the village tended to have higher *N. americanus* infection risk, and we expect this pattern to hold across seasons. Finally, alternative approaches, such as exponential random graph models, can explicitly account for network structure and may be useful for identifying network characteristics associated with infection patterns, such as degree heterogeneity across nodes. We used a simple permutation-based approach because our goal was to test infection clustering on weighted land-use networks while avoiding challenges associated with the highly right-skewed nature of edge weights in our networks.

## Conclusions

Our study offers a novel and more mechanistic understanding of hookworm transmission by linking spatial movement data with infection risk. We demonstrate how certain demographic predictors such as higher infection risk in men are related to landscape use and behavioral exposure. Our findings also demonstrate how the transmission dynamics of the zoonotic hookworm *Ancylostoma ceylanicum* differ from the human hookworm *Necator americanus*: zoonotic hookworm infection patterns have weaker relationships to human movement networks and are more likely to be related to surrounding infected dog density. Importantly, we found that relationships between human movement and hookworm infections varied based on surrounding landcover, underscoring the importance of identifying parasite species and tailoring public health interventions to local ecological and cultural contexts. By capturing exposure risk through fine-scale movement patterns across different habitats rather than static spatial data, our approach advances the spatial epidemiology of soil-transmitted helminths and supports the development of more targeted and effective control strategies.

## Supplementary Material

Supplementary Files

This is a list of supplementary files associated with this preprint. Click to download.

- SupplementaryInformation.docx
- SupportingInformationCaptions.docx

## Figures and Tables

**Figure 1:**
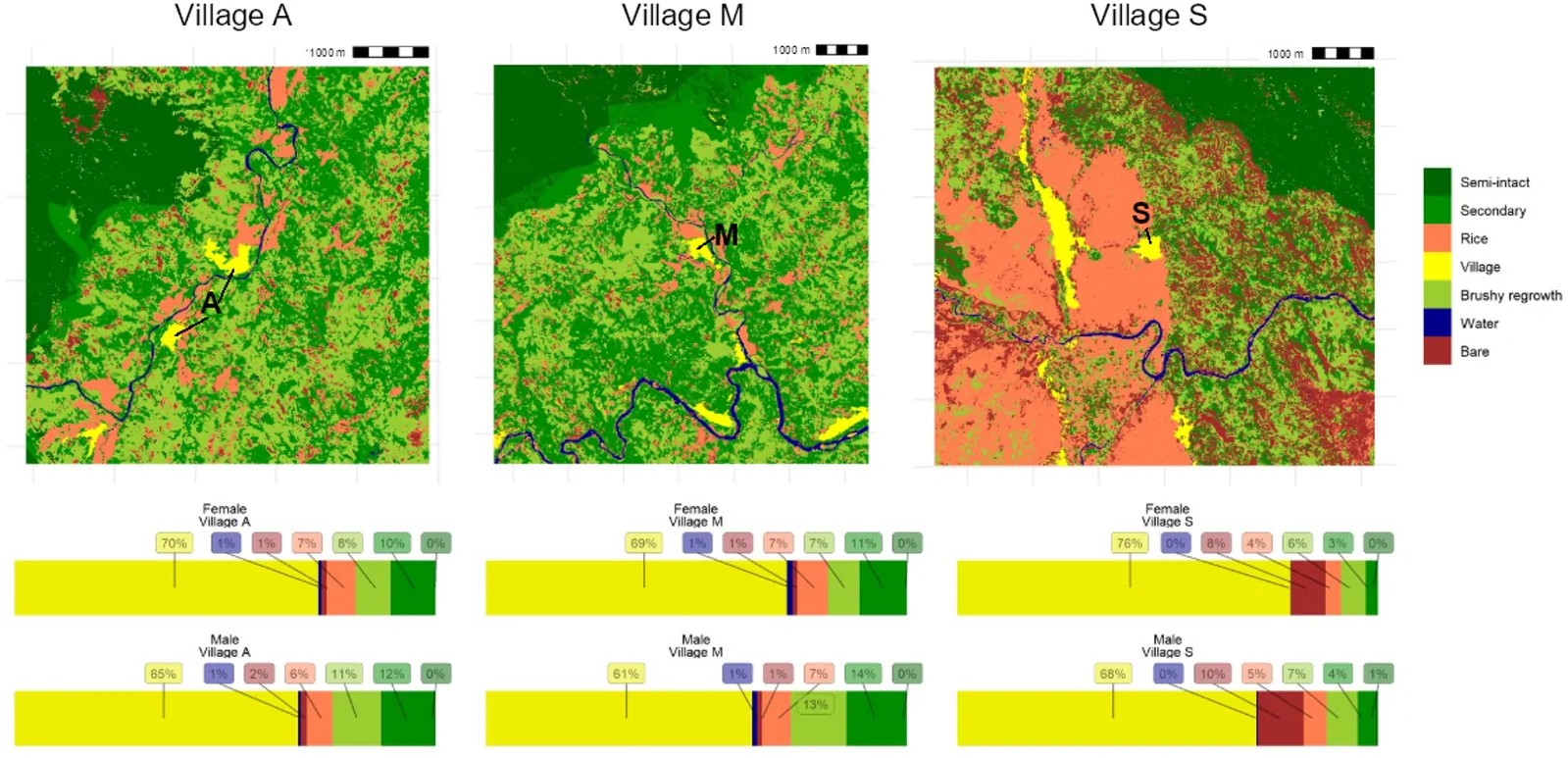
Habitat classification maps for each of the study villages. Bar charts illustrate the mean proportion of people’s utilization distribution (time spent) in each land cover type across villages and genders during typical active hours (06:00 – 19:59).

**Figure 2:**
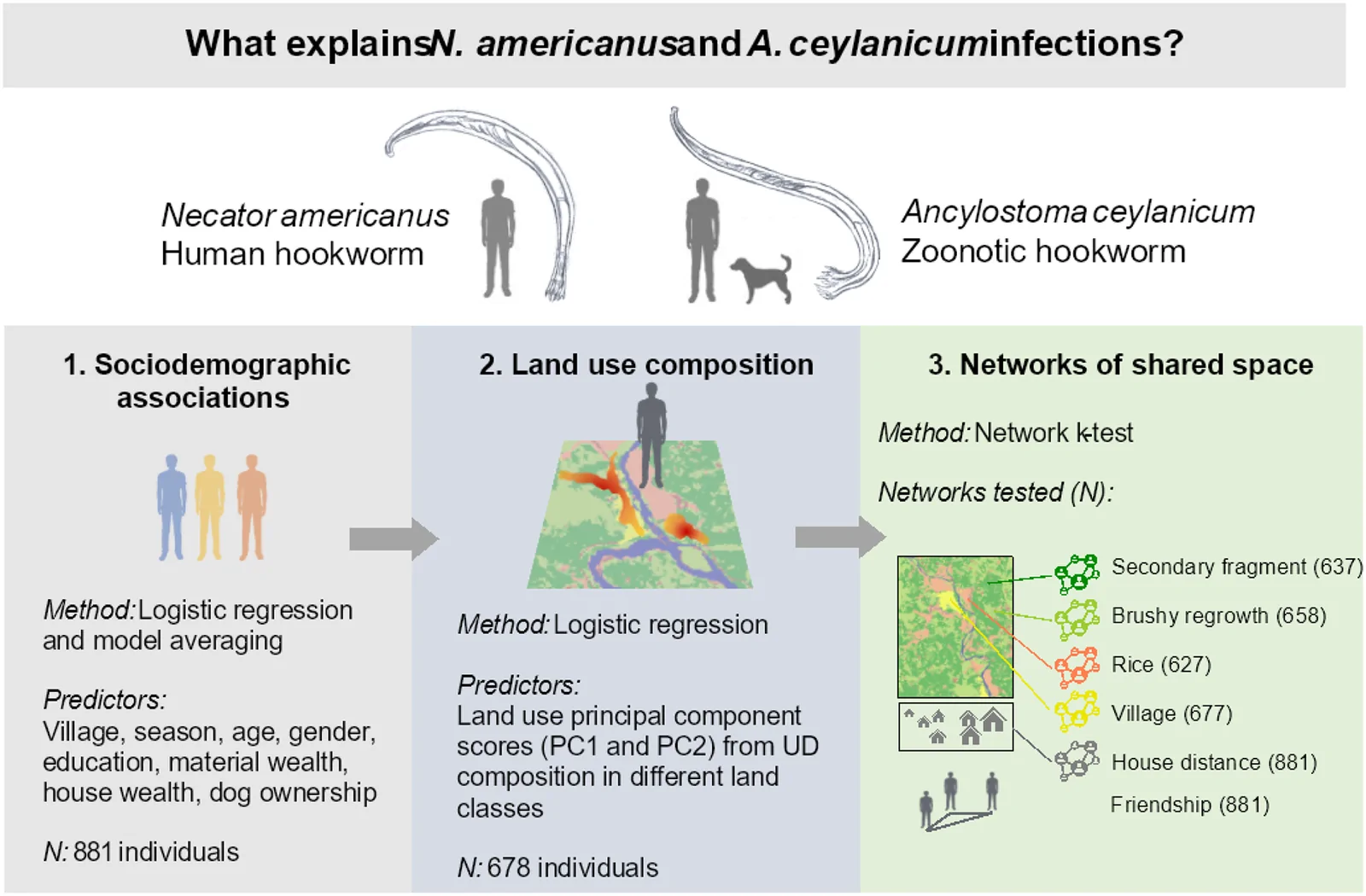
Flow chart of analyses performed in the study. We used GLMs to identify sociodemographic predictors of infection among the 881 adults in the study. The land use composition relationship to hookworm infections was assessed based on time spent in different land-cover types, which was derived from utilization distributions (UD) of the 678 individuals who wore a GPS. We tested whether infection patterns were associated with several different networks, including spatial networks built from utilization distribution intersections with landcover, a network of house distances, and the non-spatial friendship network.

**Figure 3:**
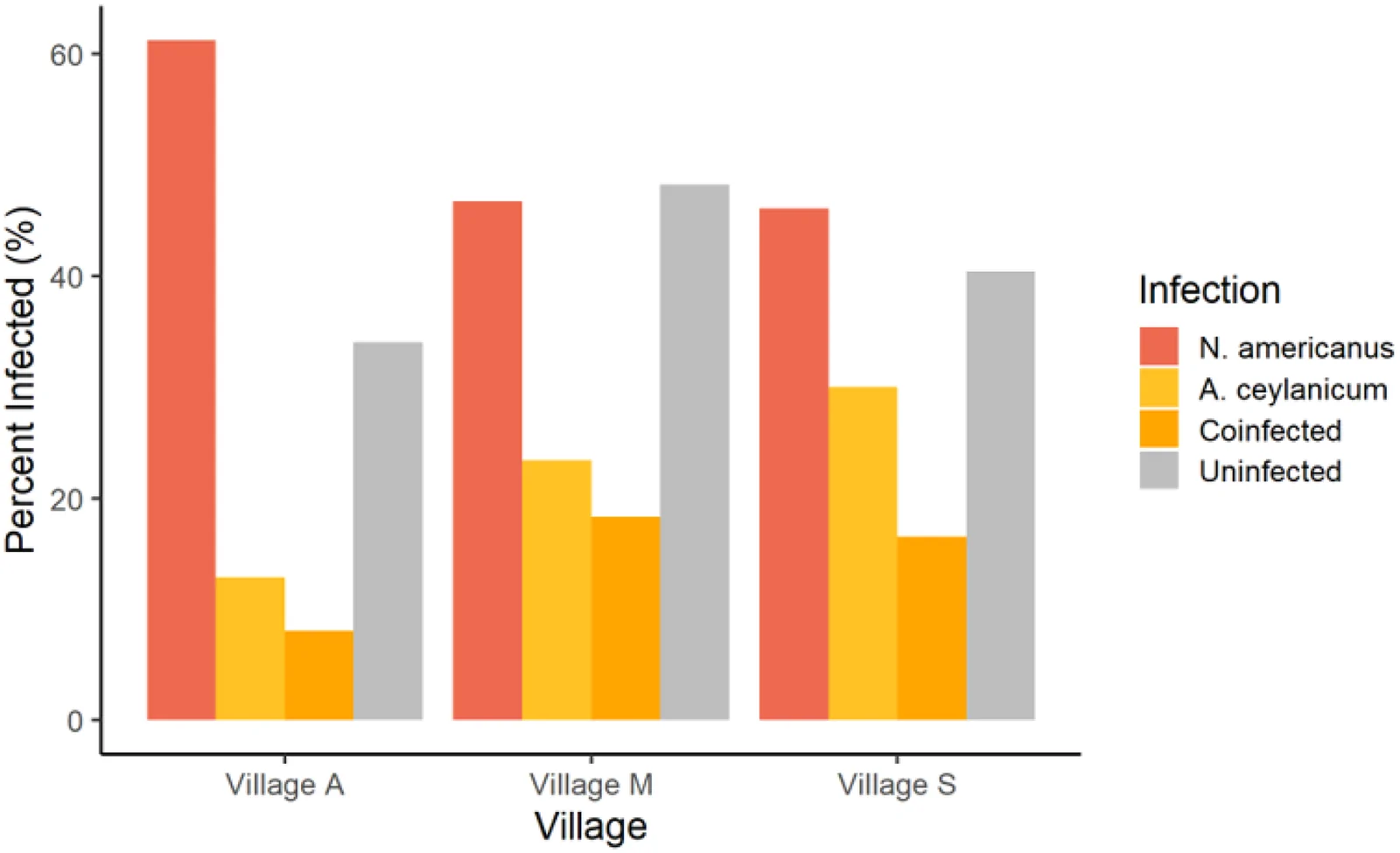
Hookworm infection and co-infection profiles for each village in the study.

**Figure 4:**
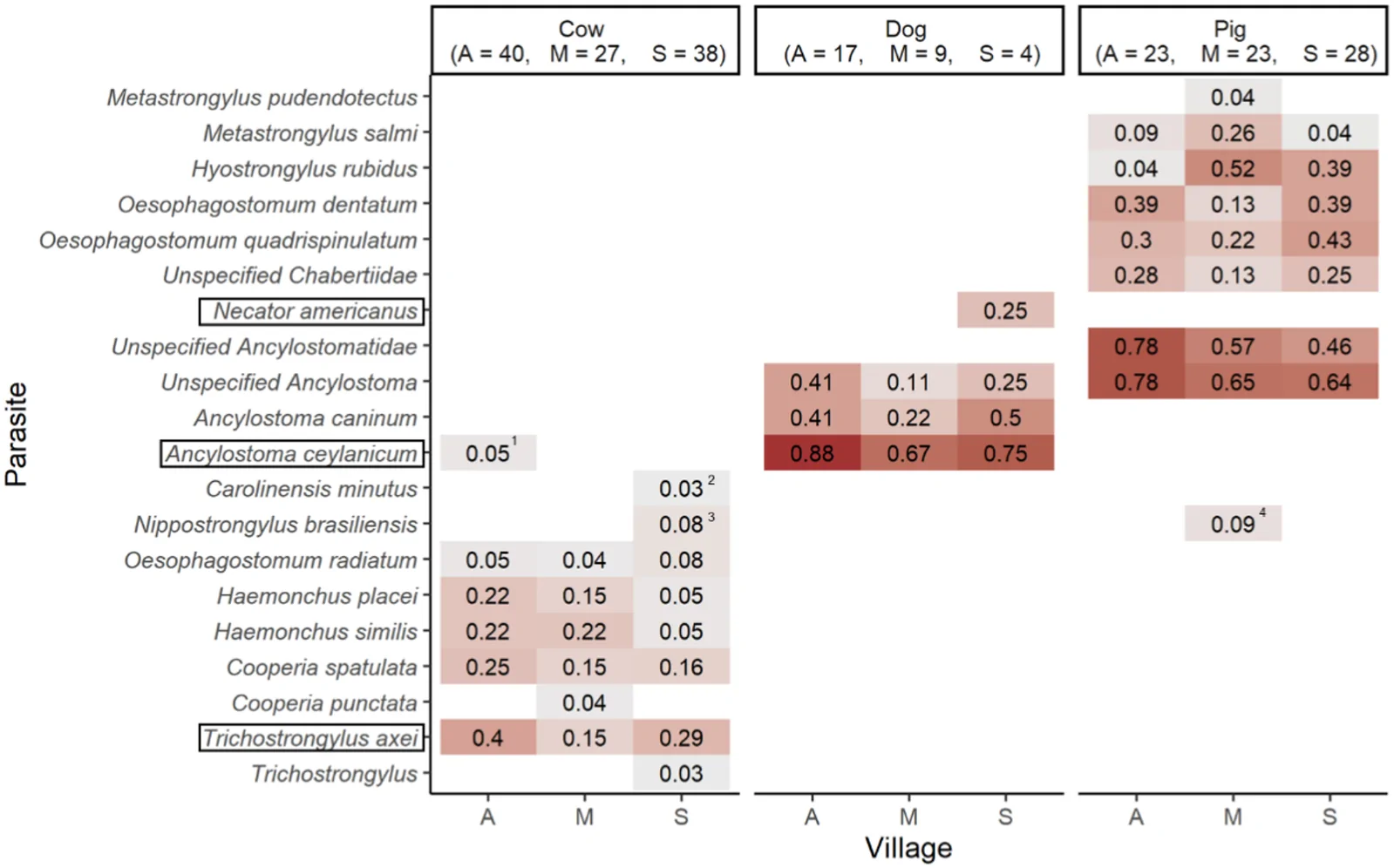
Prevalence of strongylid nematodes in cows, dogs, and pigs across the three villages. Numbers in headers indicate the sample size in each village for each species. Parasites that were also found in humans are outlined in the y-axis. There were four instances in which we found low relative read abundance (RRA<0.1) for parasite species that are atypical to a particular host and are unlikely to represent true infections, denoted by superscripts: 1. Two cows had trace *A. ceylanicum* DNA (RRA = 0.01 and 0.03); 2. One cow had trace *C. minutus* DNA (RRA = 0.01); 3. Three cows had trace *N. brasiliensis* DNA (RRA = 0.08, 0.05, and 0.03); 4. Two pigs had trace *N. brasiliensis* DNA (RRA = 0.01 and 0.01). Trace levels of *N. brasiliensis* and *C. minutus* may indicate environmental or feed contamination by peridomestic rodents. Due to logistical challenges in collecting fresh samples from domesticated animals, sample sizes are considerably smaller than for humans.

**Figure 5:**
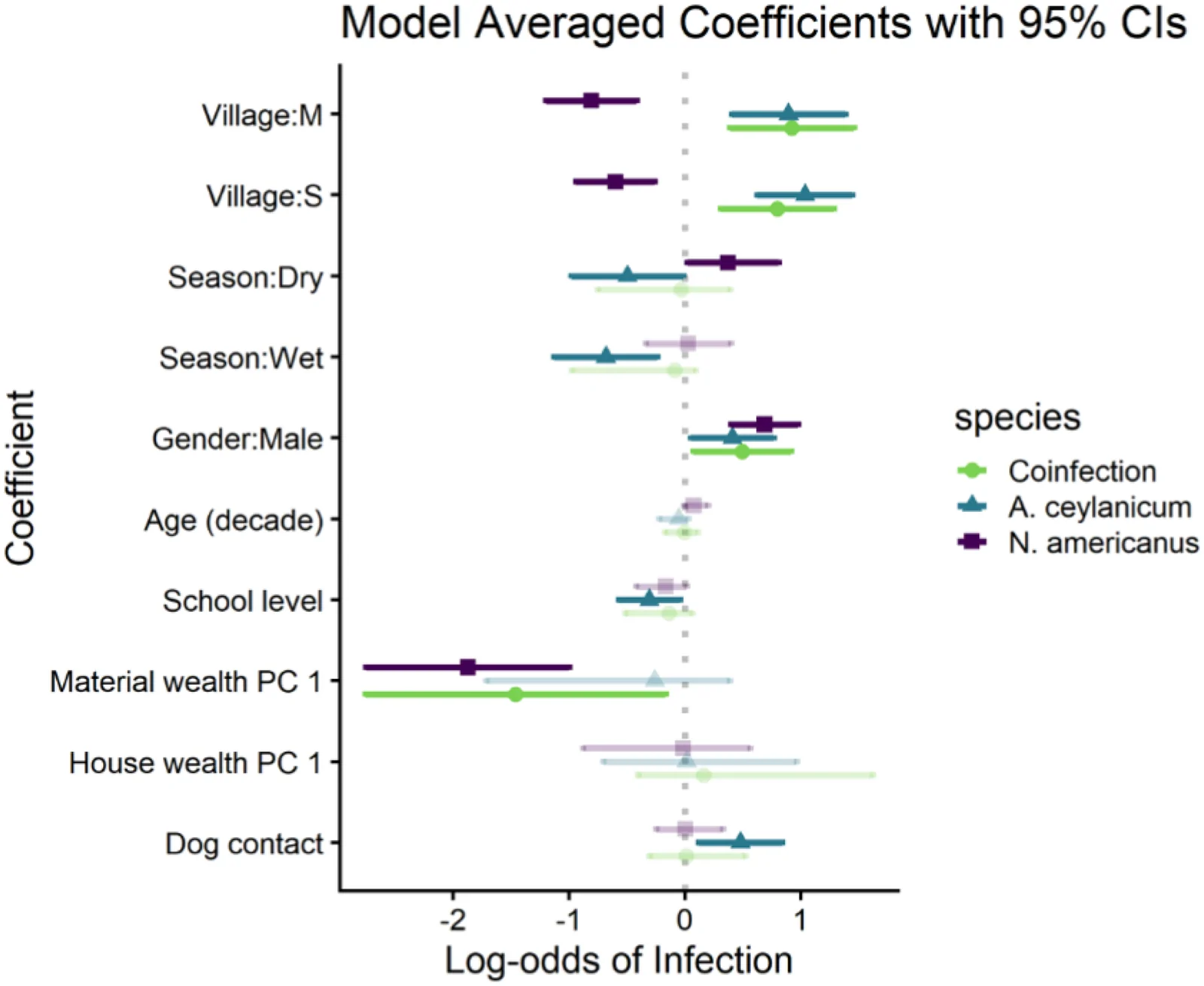
Model-averaged coefficients for each model of hookworm infection and coinfection. Reference levels for categorical predictors were Village A and Female. Points and error bars to the left of the dotted gray line indicate lower odds of infection, while those to the right indicate higher odds of infection. Points and error bars with transparency indicate no significant association.

**Figure 6:**
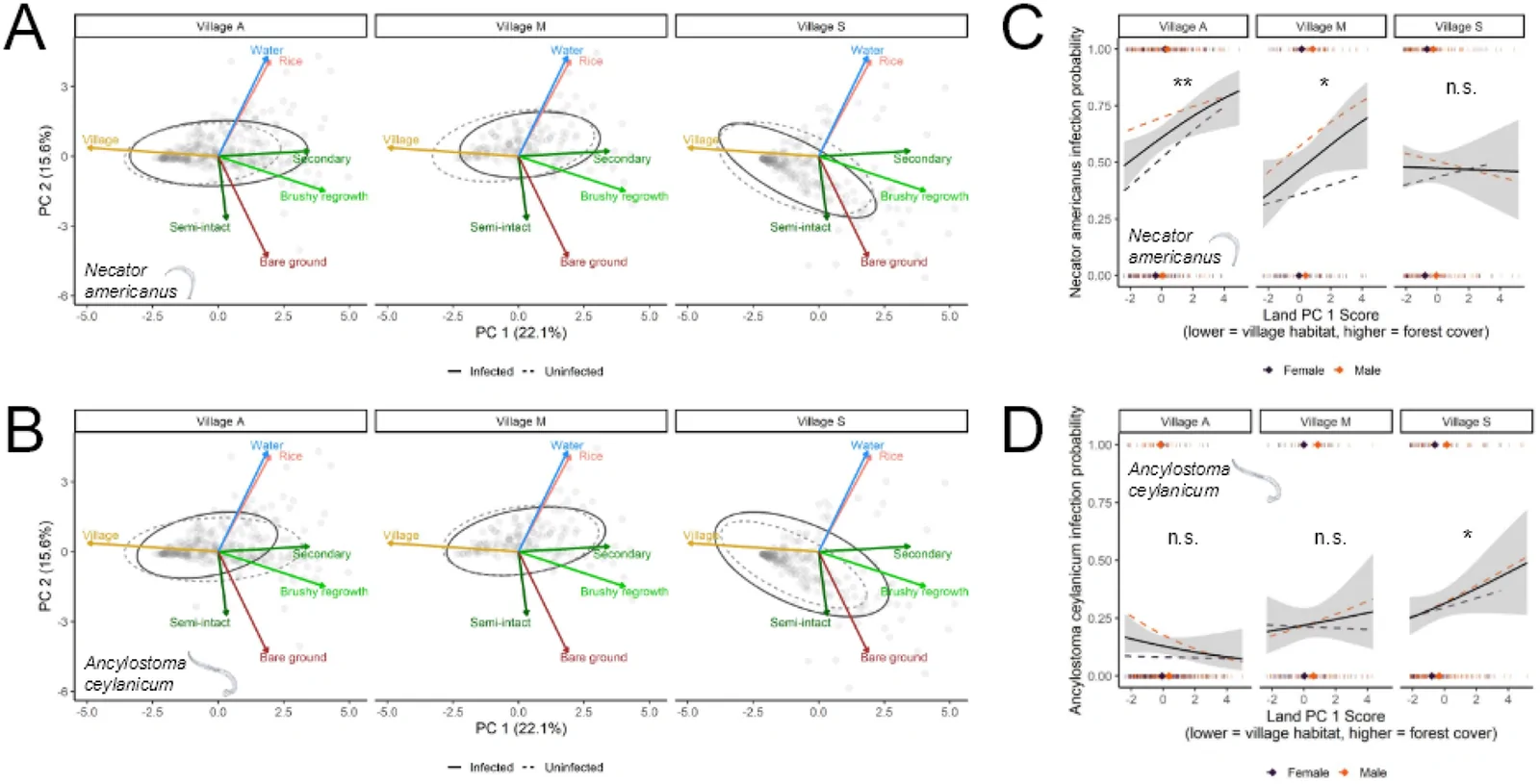
Associations between human movement and hookworm infection. A and B) Principal component analysis of land use using the proportion of utilization distributions comprised by each land cover type by individuals for all villages. Separate hulls represent 95% ellipses based on *N. americanus* infection status (panel A) and *A. ceylanicum* infection status (panel B). C and D) Relationship between PC1 scores and *N. americanus* infection probability (panel C) and *A. ceylanicum* infection (panel D) for men and women across villages. Rug plots on the top and bottom of each panel illustrate the distribution of individual PC1 scores for infected and uninfected individuals; points on the rug plots represent mean PC1 scores.

**Figure 7:**
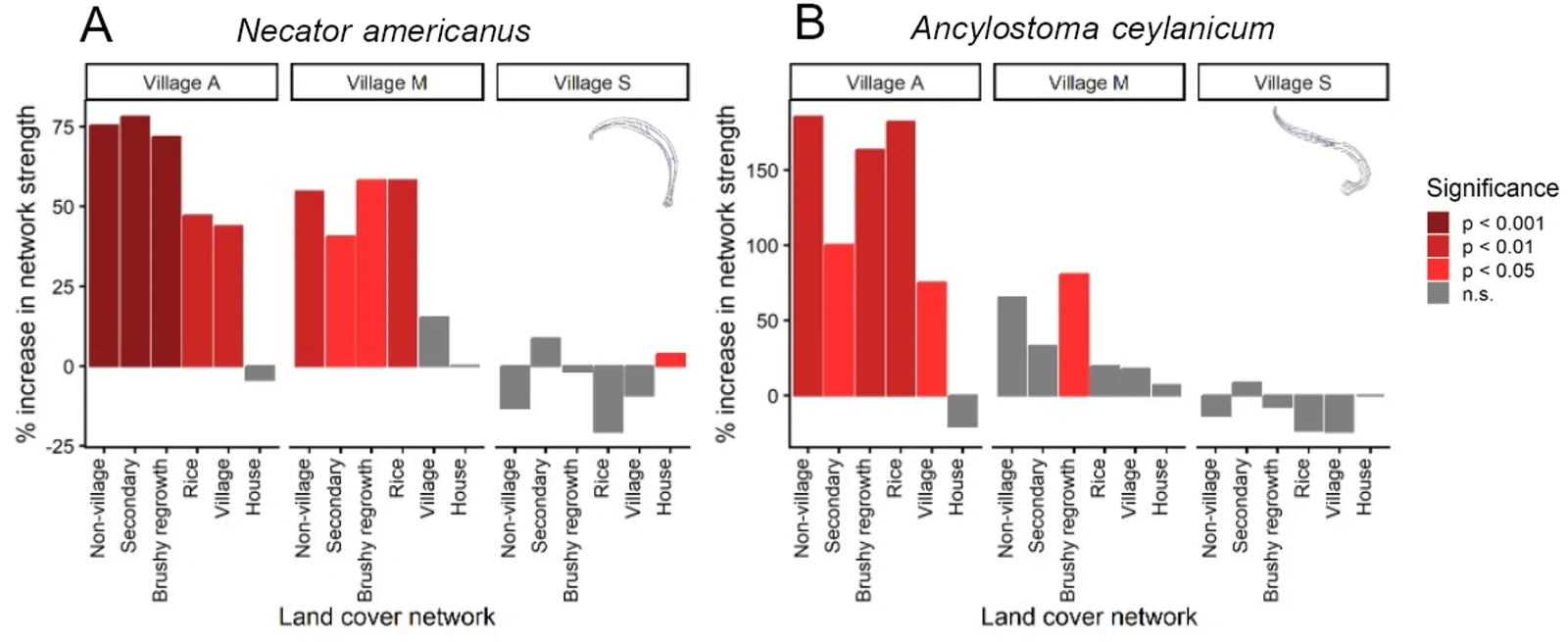
Networks of individual land use as predictors of infection with hookworm in three villages. (A) Human hookworm infection was associated with overlap in vegetated areas for two villages that had more of this type of habitat, and by house distance in the remaining village. (B) Zoonotic hookworm tended to be independent of patterns of human land use sharing, except in village A.

**Table 1:** Summary of sample sizes and mean values (± standard deviation) for demographic and movement data across the three villages.

N fecal samples	Village A	Village M	Village S
	417	197	267
Dry Season N	160	120	43
Wet Season N	132	77	128
Transitional Season N	126	-	96
Approximate proportion of village population	15%	10%	30%
N with any missing demographic data	7	14	3
% Women	49.6	49.7	41.9
Mean school level [0 = none; 1 = primary; 2 = secondary; 3 = higher]	1.64 ± 0.74	1.36 ± 0.70	1.63 ± 0.75
Mean material goods wealth PC (higher = wealthier)	0.002 ± 0.166	−0.004 ± 0.173	0.025 ± 0.176
Mean household materials wealth PC (higher = wealthier)	−0.014±0.213	−0.016 ± 0.238	0.035 ± 0.199
% Individual close contact with dogs	49.6	54.9	55.8
% Household dog ownership	25.2	35.4	41.8
% Household pig ownership	34.5	22.6	25.5
% Household cow ownership	36.3	31.3	31.0
N individuals with GPS and infection data	301	156	221
*Necator americanus* prevalence	61.4%	46.7%	46.1%
*Ancylostoma ceylanicum* prevalence	12.9%	23.4%	30.0%
Coinfection prevalence	8.13%	18.3%	16.5%

## Data Availability

Summarized data are provided in the manuscript. Due to privacy and ethical restrictions on sharing human subjects data with location information and demographic details that in combination may compromise anonymity, individual-level data are available upon request by contacting the Duke University Institutional Review Board (campusirb@duke.edu). Anonymized hookworm sequences from positive samples are available from the National Center for Biotechnology Information, BioProject ID PRJNA1496440.
